# Paleolithic diet fraction and score in post hoc data analysis of a randomized controlled trial with lifestyle interventions for abdominal obesity

**DOI:** 10.1186/s40795-025-01165-4

**Published:** 2025-10-02

**Authors:** Björn Rydhög, Pedro Carrera-Bastos, Yvonne Granfeldt, Kristina Sundquist, Tommy Jönsson

**Affiliations:** 1 Center for Primary Health Care Research, Department of Clinical Sciences Malmö, Lund University, Malmö, Sweden; 2 Department of Process and Life Science Engineering, Lund University, Lund, Sweden; 3 University Clinic Primary Care, Skåne University Hospital, Region Skåne, Malmö, Sweden; 4 Center for Primary Health Care Research, Skåne University Hospital, Jan Waldenströms gata 35, CRC, hus 28 plan 11, Malmö, 205 02 Sweden

**Keywords:** Paleolithic diet, Paleolithic diet fraction, Paleolithic diet score, Lifestyle change, Type 2 diabetes, Waist circumference

## Abstract

**Background:**

Paleolithic Diet Fraction (PDF) and Paleolithic Diet Score (PDS) are both measures of how closely a food intake conforms to a Paleolithic dietary pattern. PDF is calculated directly from an individual’s absolute food intake and PDS is an aggregated score based on an individual’s food intake relative to a population. Both are inversely associated with all-cause and cause-specific mortality, coronary events and cancer. Here, for the first time, both measures are calculated in the same population and compared in a post hoc data analysis of a two-year-long randomized controlled trial (RCT) examining effects of lifestyle changes on waist circumference.

**Methods:**

Seventy-three participants with increased waist circumference and at least one additional risk factor for cardiovascular disease were randomized to a diet based on current dietary guidelines with or without grains and with or without physical exercise or controls. Of these, this post hoc analysis included the 57 participants (36 women and 21 men, aged 31–79 years) who completed the study per protocol with dietary data. Mean daily PDF and PDS were calculated using four-day weighed food records at baseline, 12 months, and 24 months. Correlations between PDF and PDS, and their respective associations with cardiometabolic outcomes were examined.

**Results:**

The mean daily PDF for energy at baseline, 12, and 24 months was 36%, 44%, and 42%, respectively. The absolute values of PDF and PDS, and their relative changes, demonstrated moderate to strong correlations during the study (*r*_*s*_*​(*55) = 0.38-0.75, *p* < .001-0.004). No significant associations were found between changes in absolute or relative measures of PDF or PDS and changes in cardiometabolic outcomes.

**Conclusions:**

There were moderate to strong correlations between PDF and PDS and no associations between PDF or PDS and cardiometabolic outcome measures, with the latter result possibly due to only modest changes in diet and an absence of outcome effects.

**Registry:**

ClinicalTrials.gov, TRN: NCT01208558, Registration date: 24 September 2010. Retrospectively registered.

## Background

The Paleolithic diet is a dietary pattern emulating the nutritional intake of the late-Paleolithic humans, comprising fruits, vegetables, roots, tubers, lean meats, fish, seafood, eggs, and nuts, while excluding cereal grains, dairy products, legumes, refined fats, and sugar [1, 2]. The diets of recent hunter-gatherer populations are thought to most closely resemble the nutritional intake of late-Paleolithic humans. A Paleolithic diet has been hypothesized to be ideal for the prevention of chronic diseases such as obesity and type 2 diabetes [1, 2]. This hypothesis is supported by the absence of these conditions in contemporary hunter-gatherer populations and by the beneficial metabolic effects observed in Paleolithic dietary interventions among modern individuals [2–5]. Support also comes from studies examining associations between measures of how closely a diet conforms to a Paleolithic diet pattern and morbidity and mortality [6–15]. The Paleolithic Diet Fraction (PDF) and Paleolithic Diet Score (PDS) are both measures of how closely a food intake conforms to a Paleolithic dietary pattern. PDF estimates how large a portion of the absolute dietary intake stems from food groups included in the Paleolithic diet [16], and PDS is an aggregated score based on an individual’s food intake relative to a population [6].

PDFs were reported in two randomized controlled trials (RCT) on participants with ischemic heart disease or type 2 diabetes as around 80% for the Paleolithic diet and around 40% for a Mediterranean-like and diabetes diet [9, 16]. In these short, three-month intervention studies, higher PDFs were associated with improved glycemic control and healthier levels of cardiometabolic risk factors, including reduced waist circumference and body weight, as well as more favorable blood lipid levels (total cholesterol, low-density lipoprotein cholesterol, and triglycerides) [9, 16]. In addition, there was an inverse association between PDF and risk of death from all-cause as well as cause-specific mortality and incidence of coronary events in the Malmö Diet and Cancer Study (MDCS), a prospective cohort study of 24,104 participants aged 44–74 years (mean age 57 years (SD 10 years), 63% women) [10].

The PDS, developed by Whalen et al. (2014), has been estimated in prospective cohort diet studies [6, 7, 13, 14, 17, 18] and is described in detail elsewhere [6]. Briefly, PDS is based on habitual food consumption information. Each study participant is assigned a quintile rank (a score from 1 to 5) based on the sex-specific distribution of intake for each food group included in the PDS, as shown in Table 1. An inverse association between PDS and risk of death from all causes was observed in two large prospective cohort studies:

**Table 1:** Constituents and construction of Paleolithic Diet Fraction and Paleolithic Diet Score

Paleolithic Diet Fraction	Paleolithic Diet Score		
	Intake Category	Scoring	Components
*Paleolithic food groups* Vegetables Fruits Potatoes Egg Meat Fish, shellfish Water	Highest intake “best”	No. of points assigned to each quintile = quintile rank (e.g., highest and lowest quintiles scored + 5 and + 1 points, respectively)	Vegetables Fruits Fruit and vegetable Diversity^a^ Lean meats^b^ Fish Nuts Calcium^c^
*Non-Paleolithic food groups* Meat products Cereal grains Rice Legumes Dairy foods Oil, butter, fat Soup, sauce, filling salad Candy, snacks, ice cream, soda Jam Spirits Wine Beer Sugar-sweetened beverages Juice Tea, Coffee	Lowest intake “best”	No. of points assigned to each quintile = reverse quintile rank (e.g., highest and lowest quintiles scored + 1 and + 5 points, respectively)	Red and processed meat^d^ Sodium Dairy foods Grains and starches Baked goods^e^ Sugar-sweetened beverages Alcohol

*Note*. Summary of the constituents and construction of the Paleolithic Diet Fraction (PDF) and Paleolithic Diet Score (PDS) in this study. Mean daily dietary intake was calculated from four-day weighed food records. PDF was calculated as the fraction of the mean daily dietary intake of Paleolithic food groups divided by the mean daily dietary intake of all food groups. For PDS, participants were assigned a sex-specific quintile rank (and a corresponding score from 1 to 5) of intake for each of 14 food components (range of possible scores 14–70)

^a^ Fruit and vegetable diversity was calculated as mean daily diversity scores by summing the total number of intakes from different fruits and vegetables

^b^ Lean meats equated with the Paleolithic food group Meat

^c^ Intake of calcium from sources other than dairy foods was calculated as residuals from the linear regression of total calcium intake (mg/day) on dairy-food intake

^d^ Red and processed meat equated with the non-Paleolithic food group Meat products

^e^ Baked goods equated with the non-Paleolithic food groups Candy, snacks, ice cream, soda and Jam

The REGARDS study, including 21,423 participants from all contiguous 48 U.S. states aged ≥ 45 years (mean age 66 years (SD 9 years), 56% women) [7], and the Moli-sani Study, including 22,849 participants in Italy aged ≥ 35 years (mean age 55 years (SD 12 years), 52% women) [13].

PDF and PDS have never been calculated within the same population, making it unclear how these different measures of how closely a diet conforms to a Paleolithic diet pattern relate to each other. Hypothetically, there should be a correlation between the two measures and a similarity of association with changes in cardiometabolic outcomes. The aim of this study is to calculate both PDF and PDS in the same population and estimate their correlation with each other and their association with cardiometabolic outcomes in a post hoc data analysis of a two-year-long randomized controlled trial of 73 participants (47 women and 26 men aged 23–79 years), examining effects on waist circumference of a diet based on the Swedish Food Agency’s dietary guidelines with or without cereal grains and with or without physical exercise [19]. The original study found no significant associations between diet and exercise interventions and changes in waist circumference or any other cardiometabolic outcome, despite the greatest group difference being more than double than estimated in the pre-study power calculation [19]. The non-significant results could be explained by too few participants and a greater than expected outcome variability [19].

## Materials and methods

The study protocol (LU 2010/332) was approved by the Regional Ethical Board in Lund. All participants gave written informed consent.

### Study design and population

**Fig. 1:**
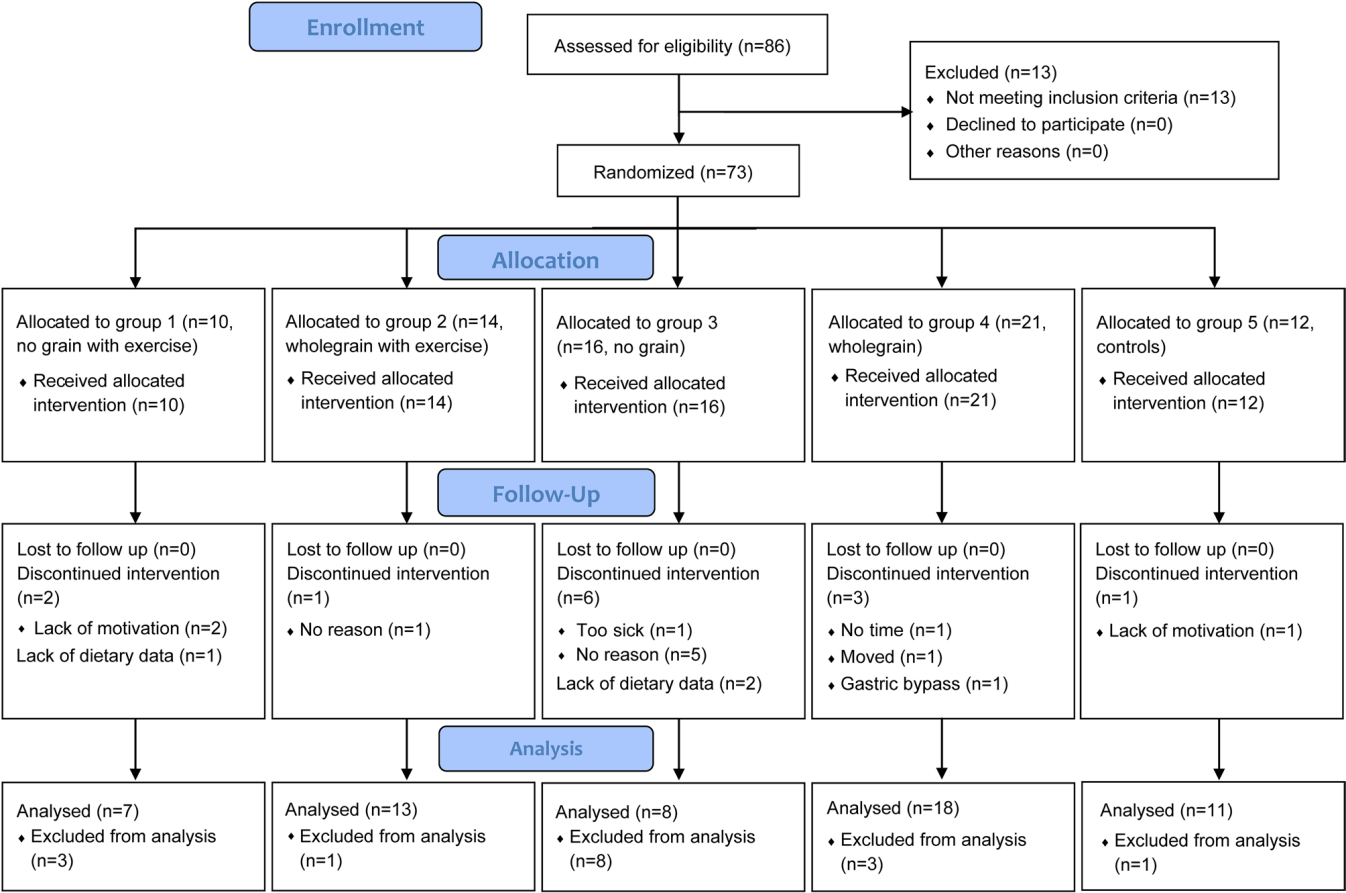
CONSORT flow diagram of participants

The participants of this two-year-long RCT with a factorial design were adult individuals belonging to a public Swedish primary health care center in the university town of Lund, Sweden, with increased waist circumference and at least one additional risk factor for cardiovascular disease. Eligible participants were randomly allocated to eat a healthy diet based on the Swedish Food Agency’s dietary guidelines for people affected by overweight or obesity with or without grains and with or without physical exercise, or to a control group with follow-up only [19]. Details of the interventions, the cohort and measurements are described elsewhere [19]. Briefly, in the “no grain” diet (diet A, groups 1 and 3), all types of grain should be avoided (bread, pasta, cereals, porridge, pies, crackers, whole grain and rice), and in the “whole grain” diet (diet B, groups 2 and 4), refined cereal grain products were replaced by corresponding whole grain cereal products. Groups 1 and 2 were also allocated to physical exercise (pedometers and two initial months of weekly structured exercise followed by a written prescription of physical activity). Group 5 was the control group receiving no intervention. A total of 73 participants (47 women and 26 men, aged 23–79 years) started the trial. Thirteen participants discontinued the intervention, and an additional three participants lacked dietary data. The study was thus completed per protocol with dietary data by 57 participants (36 women and 21 men), all of whom were included in this post hoc analysis (Fig. 1).

The primary outcome was waist circumference, and secondary outcomes were body weight, systolic and diastolic blood pressure, total and HDL cholesterol, fasting plasma glucose, and HbA1c, all measured at baseline and after 3, 6, 12, and 24 months. Standing height was only measured at baseline. Body fat percentage, subcutaneous fat, physical activity, food intake, and change in medication for diabetes and hypertension were all measured and recorded at baseline and after 12 and 24 months.

Food intake was assessed using four-day weighed food records filled out by the participants during the study. Participants were instructed to weigh all food items using a tare-enabled electronic kitchen scale. When weighing was not possible, quantities were estimated using alternative units (e.g., deciliters, tablespoons, teaspoons, or centimeters). Although less precise, these estimates represent a minor limitation, as most items were weighed, and remaining entries were converted to grams by YG, who has extensive experience in translating food records. Nutrient intake was calculated based on the reported food intakes using the contemporary Food Database of the Swedish Food Agency [20].

### PDF and PDS

PDFs for energy and weight were calculated from the reported food intake as the fraction between the mean daily intake of Paleolithic food groups and the mean daily intake of all food groups (Table 1). The PDS was also constructed from the reported food intake, similarly as previously described [6]. Briefly, each participant was assigned a quintile rank (a score from 1 to 5) based on the sex-specific distribution of intake by weight for each food group included in the score. Generally, more points were assigned for higher intakes of foods considered characteristic of the Paleolithic diet pattern, and fewer points were assigned for higher intakes of foods considered uncharacteristic of the pattern. The points for each of the 14 food groups composing the diet score were then summed, so that the final PDS could range from 14 to 70, as previously described (Table 1).

### Statistical analysis

The SPSS statistical computer package (version 29.0; IBM Corporation, Armonk, NY, USA) was used for all statistical analyses. Data normality was assessed with Normal Q-Q plot and Shapiro-Wilk test. Groups were compared using the X^2^, Fisher’s exact, one-way ANOVA, or Kruskal-Wallis test, as appropriate, followed by Bonferroni-adjusted *post hoc* tests (X^2^, Tukey, Mann-Whitney). Spearman correlation coefficients were calculated between the PDF for energy and weight and PDS. Generalized Estimating Equations were used to assess associations between relative and absolute changes in PDF for weight and energy and PDS, and relative and absolute changes in outcome measures from 0 to 12 months and 12–24 months. Models accounted for within-subject correlations using an appropriate working correlation structure and adjusted for sex, age, and training intervention. Assumptions included correct specification of the mean model, missing data, and sufficient sample size for valid inference. Fasting plasma glucose, HbA1c, systolic blood pressure, and diastolic blood pressure were excluded from the analysis because the participants changed their medication for diabetes and hypertension at an unknown time during the study. A sensitivity analysis, in the form of an intention-to-treat analysis, was conducted including all 73 randomized participants who started the study, regardless of whether they completed the study or lacked dietary data. All statistical tests were two-sided, and statistical significance was set at *p* < .05.

## Results

**Fig. 2:**
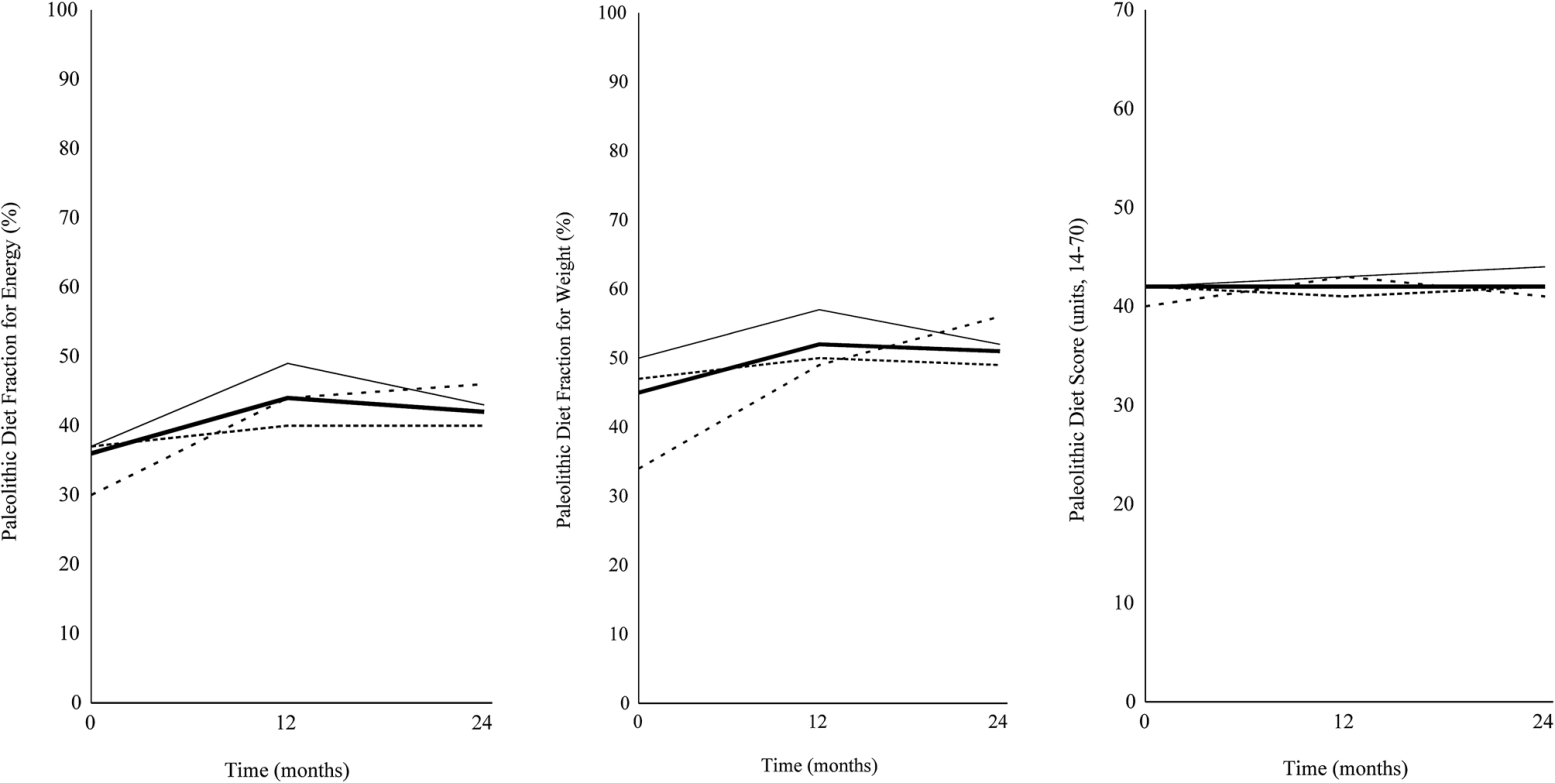
Mean Paleolithic Diet Fraction for Energy and Weight, and Paleolithic Diet Score at Baseline, 12 Months, and 24 Months. *Note.* Mean Paleolithic Diet Fraction for energy and weight, and Paleolithic Diet Score at baseline, 12 months, and 24 months, based on all 57 participants who completed the study per protocol (from 73 randomized participants) with complete dietary data. Diet **A** (No grain, thin solid line), Diet **B** (Whole grain, shortly spaced dashed line), controls (widely spaced dashed line), and all participants (thick solid line)

**Fig. 3:**
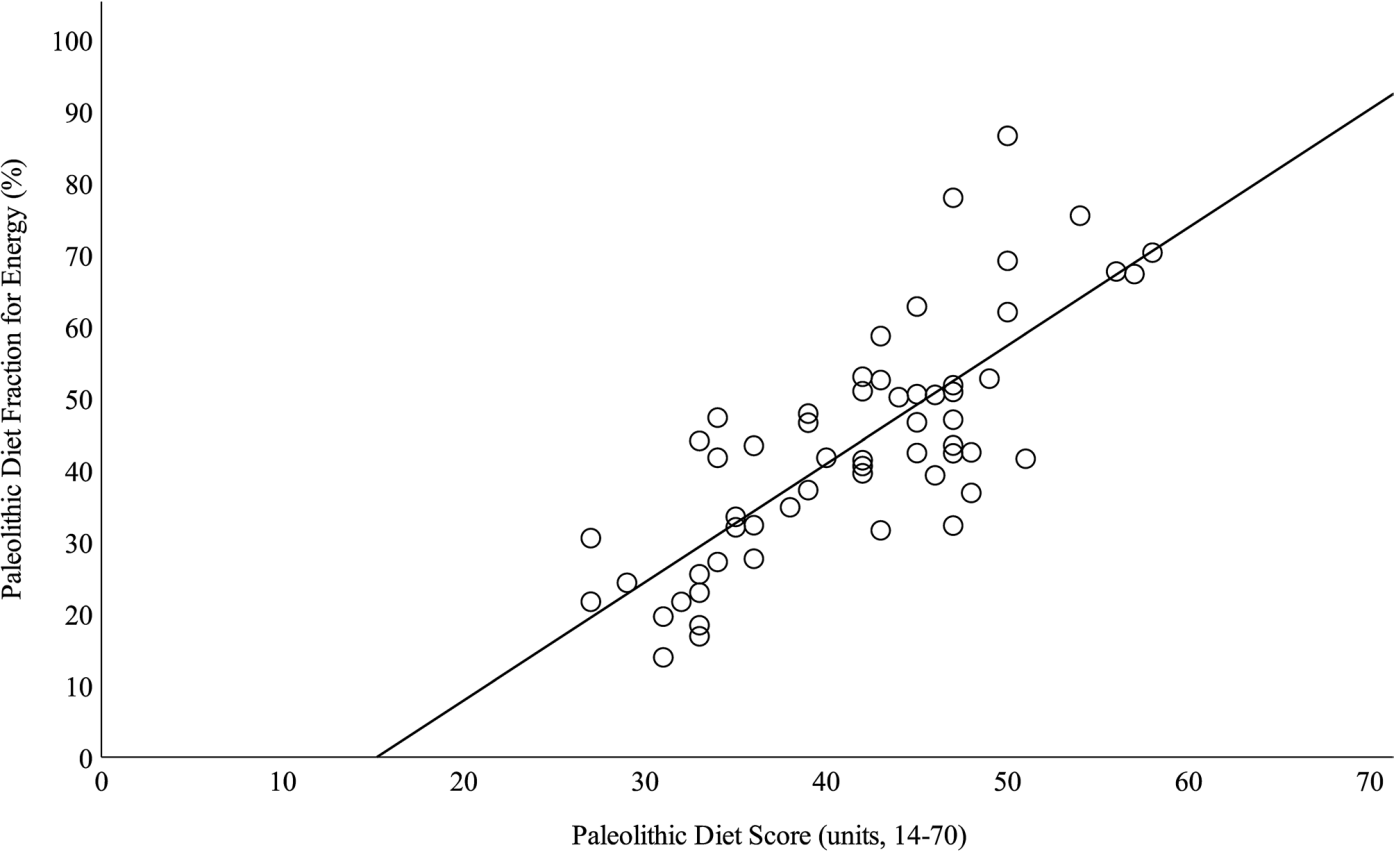
Correlation Between Paleolithic Diet Fraction for Energy and Paleolithic Diet Score at 12 Months. *Note.* Correlation between Paleolithic Diet Fraction for energy and Paleolithic Diet Score at 12 months, based on all 57 participants who completed the study per protocol (from 73 randomized participants) with complete dietary data. The solid line represents the linear regression equation (y = − 25.05 + 1.65 * x, R² = 0.609, *p* < .001)

Baseline characteristics of the 57 participants included in this post hoc analysis, PDF for energy and weight, PDS and cardiometabolic outcomes at baseline, 12 months, and 24 months are presented in Table 2. Mean PDF for energy and weight, and PDS at baseline, 12 months, and 24 months are shown in Fig. 2. The mean PDFs for the macronutrients and micronutrients at baseline, 12 months, and 24 months are presented in Table 3. The correlations between PDF for energy and weight and PDS at baseline, 12 months, and 24 months are presented in Table 4. The correlation between PDF for energy and PDS at 12 months is shown in Fig. 3.

**Table 2:** Baseline characteristics and outcomes during study

Variable	Time (months)	All	Diet A (No Grain)	Diet B (Whole Grain)	Controls	*p*
Participants, *n*		57	15	31	11	0.19
Male/Female, *n*		21/36	5/10	11/20	5/6	0.80
Height, cm *M (SD)*	0	171 (9)	169 (11)	172 (9)	170 (7)	0.45
Age, years *M (SD)*	0	61 (8)	57 (10)	63 (7)	62 (8)	0.09
PDS, units (14–70) *M (SD)*	0	42 (6)	42 (7)	42 (6)	40 (5)	0.49
	12	42 (8)	43 (7)	41 (8)	43 (9)	0.69
	24	42 (7)	44 (6)	42 (6)	41 (10)	0.36
PDF for Weight, % *M (SD)*	0	45 (15)	50 (13)a	47 (14)b	34 (15)ab	0.01
	12	52 (18)	57 (18)	50 (17)	49 (19)	0.38
	24	51 (15)	52 (17)	49 (13)	56 (17)	0.45
PDF for Energy, % *M (SD)*	0	36 (12)	37 (13)	37 (12)	30 (8)	0.20
	12	44 (16)	49 (19)	40 (13)	44 (18)	0.20
	24	42 (12)	43 (14)	40 (9)	46 (18)	0.76
Waist Circumference, cm *M (SD)*	0	106 (11)	107 (11)	105 (10)	108 (14)	0.81
	12	104 (11)	104 (12)	104 (10)	104 (13)	1.0
	24	106 (11)	108 (15)	105 (9)	106 (13)	0.71
Weight, kg *M (SD)*	0	92 (15)	92 (17)	93 (14)	90 (18)	0.88
	12	90 (14)	90 (16)	91 (13)	88 (15)	0.77
	24	90 (14)	91 (17)	91 (12)	89 (16)	0.93
Total Cholesterol, mmol/L *M (SD)*	0	5.3 (1.2)	5.0 (0.9)	5.5 (1.3)	5.0 (1.2)	0.40
	12	5.1 (1.2)	4.7 (0.8)	5.4 (1.3)	5.0 (1.3)	0.13
	24	5.0 (1.2)	4.6 (0.8)	5.2 (1.3)	4.9 (1.3)	0.33
HDL, mmol/L *M (SD)*	0	1.3 (0.4)	1.2 (0.3)	1.4 (0.4)	1.3 (0.4)	0.60
	12	1.4 (0.5)	1.3 (0.4)	1.5 (0.5)	1.4 (0.4)	0.64
	24	1.4 (0.5)	1.3 (0.3)	1.5 (0.6)	1.4 (0.4)	0.38
Non-HDL, mmol/L *M (SD)*	0	3.9 (1.1)	3.7 (0.9)	4.1 (1.2)	3.7 (1.1)	0.44
	12	3.7 (1.1)	3.3 (0.9)	3.9 (1.2)	3.6 (1.2)	0.23
	24	3.5 (1.1)	3.3 (0.7)	3.6 (1.3)	3.5 (1.1)	0.71
Body Fat, % *M (SD)*	0	38 (7)	39 (8)	38 (8)	36 (4)	0.29
	12	38 (7)	38 (8)	38 (8)	36 (6)	0.62
	24	38 (8)	41 (8)	38 (8)	36 (6)	0.34
Visceral Fat, % *M (SD)*	0	13 (4)	13 (5)	13 (3)	14 (6)	0.89
	12	13 (4)	13 (4)	13 (3)	14 (6)	0.79
	24	13 (4)	14 (4)	13 (3)	15 (6)	0.67
Biceps Skinfold, mm *M (SD)*	0	18 (7)	19 (6)	18 (8)	18 (7)	0.96
	12	18 (6)	17 (6)	19 (7)	17 (7)	0.72
	24	18 (6)	18 (6)	17 (6)	18 (8)	0.76
Triceps Skinfold, mm *M (SD)*	0	28 (9)	30 (9)	27 (9)	26 (9)	0.53
	12	28 (8)	29 (8)	29 (9)	25 (10)	0.41
	24	29 (9)	30 (10)	29 (8)	26 (8)	0.53
Subscapular Skinfold, mm *M (SD)*	0	33 (9)	34 (12)	32 (8)	35 (5)	0.60
	12	33 (8)	35 (10)	33 (8)	32 (6)	0.62
	24	34 (7)	35 (7)	34 (7)	34 (6)	0.83
Supra-iliac Skinfold, mm *M (SD)*	0	27 (8)	28 (9)	26 (8)	27 (8)	0.78
	12	27 (8)	29 (9)	26 (7)	28 (8)	0.41
	24	28 (8)	28 (9)	27 (8)	30 (8)	0.72
SED, min/d *M (SD)*	0	590 (81)	597 (91)	576 (82)	620 (58)	0.28
	12	571 (92)	609 (79)a	540 (91)a	612 (84)	0.02
	24	586 (75)	597 (70)	573 (79)	609 (67)	0.31
MVPA, min/d *M (SD)*	0	27 (18)	25 (19)	27 (18)	30 (18)	0.79
	12	30 (23)	35 (25)	26 (21)	34 (24)	0.36
	24	26 (20)	21 (18)	28 (22)	24 (20)	0.81

*Note*. Baseline characteristics and outcomes at baseline, 12 months and 24 months of participants by diet allocation, based on all 57 participants who completed the study per protocol (from 73 randomized participants) with complete dietary data. Group comparisons were conducted using a chi-square test, one-way ANOVA, or Kruskal-Wallis H test, as appropriate. Means sharing the same subscript are significantly different from each other at the *p* < .05 level

PDS = Paleolithic Diet Score, PDF = Paleolithic Diet Fraction, HDL = high-density lipoprotein cholesterol, SED = Sedentary Time, MVPA = Moderate and Vigorous Physical Activity

**Table 3:** Paleolithic Diet Fraction for macro- and micronutrients during the study

Variable	Time (months)	All	Diet A (No Grain)	Diet B (Whole Grain)	Controls	*p*
Protein, *M (SD)*	0	52 (14)	53 (17)	52 (13)	49 (12)	NS
	12	57 (16)	62 (19)	54 (15)	58 (15)	NS
	24	56 (14)	57 (14)	55 (14)	58 (15)	NS
Fat, *M (SD)*	0	36 (16)	36 (18)	37 (16)	33 (11)	NS
	12	44 (19)	47 (20)	41 (17)	45 (25)	NS
	24	43 (16)	47 (13)	40 (14)	46 (24)	NS
Carbohydrate, *M (SD)*	0	28 (11)	29 (10)_a_	30 (13)_b_	20 (7)_ab_	0.031
	12	35 (18)	45 (24)_a_	31 (14)_a_	33 (16)	0.045
	24	33 (15)	32 (18)	32 (11)	39 (20)	NS
Fibre, *M (SD)*	0	45 (16)	46 (17)_a_	49 (13)_b_	32 (12)_ab_	0.011
	12	53 (20)	69 (21)_ab_	46 (16)_a_	50 (18)_b_	0.003
	24	47 (16)	47 (16)	44 (14)	51 (22)	NS
Salt, *M (SD)*	0	47 (16)	47 (16)	51 (17)	39 (15)	NS
	12	54 (17)	58 (17)	51 (17)	56 (15)	NS
	24	53 (15)	50 (14)	52 (15)	59 (17)	NS
Ash, *M (SD)*	0	49 (13)	50 (14)	51 (13)	41 (12)	NS
	12	55 (15)	59 (15)	53 (15)	55 (15)	NS
	24	53 (13)	52 (13)	52 (13)	58 (15)	NS
Water, *M (SD)*	0	53 (16)	57 (14)	55 (16)	43 (13)	0.043
	12	59 (18)	63 (18)	58 (19)	57 (17)	NS
	24	59 (16)	58 (18)	58 (15)	62 (15)	NS
Alcohol, *M (SD)*	0	30 (42)	26 (44)	28 (42)	40 (44)	NS
	12	37 (46)	37 (48)	34 (46)	42 (44)	NS
	24	25 (40)	3 (11)_a_	31 (42)	42 (42)_a_	0.036
Monosaccharides, *M (SD)*	0	55 (12)	63 (20)_a_	58 (21)_b_	38 (19)_ab_	NS
	12	60 (21)	62 (24)	60 (17)	59 (25)	NS
	24	60 (21)	62 (24)	60 (17)	59 (25)	NS
Disaccharides, *M (SD)*	0	27 (17)	30 (14)	28 (19)	21 (12)	NS
	12	33 (20)	38 (21)	31 (20)	31 (18)	NS
	24	33 (21)	36 (22)	31 (19)	34 (26)	NS
Sucrose, *M (SD)*	0	38 (24)	42 (23)	41 (25)	25 (16)	NS
	12	46 (27)	52 (28)	45 (28)	38 (24)	NS
	24	45 (27)	47 (30)	45 (26)	44 (29)	NS
Wholegrain, *M (SD)*	0	5 (26)	4 (8)	6 (15)	0 (1)	0.032
	12	11 (27)	21 (39)	7 (17)	9 (30)	NS
	24	8 (26)	14 (35)	1 (5)	19 (40)	NS
Sum of saturated fatty acids, *M (SD)*	0	28 (14)	28 (15)	28 (16)	26 (9)	NS
	12	36 (19)	39 (20)	34 (17)	36 (22)	NS
	24	35 (17)	35 (13)	33 (16)	40 (24)	NS
Fatty acids 4:0–10:0, *M (SD)*	0	4 (5)	4 (5)	4 (4)	6 (7)	NS
	12	11 (14)	9 (11)	12 (16)	11 (11)	NS
	24	8 (11)	7 (7)	6 (8)	13 (21)	NS
Fatty acids 12:0, *M (SD)*	0	5 (6)	6 (9)	4 (5)	5 (5)	NS
	12	12 (17)	13 (13)	13 (20)	10 (11)	NS
	24	8 (13)	9 (11)	7 (13)	10 (18)	NS
Fatty acids 14:0, *M (SD)*	0	16 (10)	17 (13)	15 (10)	16 (6)	NS
	12	24 (18)	26 (22)	24 (16)	24 (18)	NS
	24	21 (17)	21 (13)	18 (13)	29 (26)	NS
Fatty acids 16:0, *M (SD)*	0	32 (15)	32(16)	33(17)	30 (9)	NS
	12	40 (19)	44 (20)	38 (18)	41 (24)	NS
	24	40 (17)	42 (13)	38 (16)	44 (24)	NS
Fatty acids 18:0, *M (SD)*	0	33 (17)	34 (18)	34 (18)	31 (12)	NS
	12	40 (19)	42 (21)	38 (16)	41 (23)	NS
	24	40 (19)	41 (17)	39 (18)	45 (24)	NS
Fatty acids 20:0, *M (SD)*	0	16 (22)	20 (27)	12 (19)	22 (20)	NS
	12	24 (26)	29 (32)	22 (24)	23 (24)	NS
	24	19 (25)	17 (22)	18 (22)	23 (35)	NS
Sum of monounsaturated fatty acids, *M (SD)*	0	40 (18)	39 (21)	42 (17)	36 (14)	NS
	12	47 (20)	51 (19)	45 (18)	47 (26)	NS
	24	48 (17)	55 (13)	45 (16)	49 (24)	NS
Fatty acids 16:1, *M (SD)*	0	57 (23)	54 (27)	56 (20)	61 (25)	NS
	12	63 (24)	67 (23)	60 (24)	66 (27)	NS
	24	66 (21)	73 (11)	62 (23)	66 (27)	NS
Fatty acids 18:1, *M (SD)*	0	38 (18)	38 (21)	40 (17)	34 (13)	NS
	12	45 (19)	50 (18)	43 (18)	45 (25)	NS
	24	47 (17)	54 (14)	43 (16)	52 (27)	NS
Sum of polyunsaturated fatty acids, *M (SD)*	0	46 (19)	46 (21)	48 (17)	40 (20)	NS
	12	53 (21)	57 (20)	50 (19)	55 (30)	NS
	24	51 (18)	58 (13)	47 (15)	52 (27)	NS
Sum of n-6 fatty acids, *M (SD)*	0	44 (35)	38 (32)	50 (37)	37 (34)	NS
	12	47 (36)	64 (36)	40 (35)	45 (35)	NS
	24	42 (34)	53 (32)	35 (32)	47 (40)	NS
Fatty acids 18:2, *M (SD)*	0	40 (19)	40 (19)	43 (18)	35 (20)	NS
	12	48 (21)	53 (20)	45 (19)	51 (29)	NS
	24	45 (19)	51 (17)	42 (16)	47 (28)	NS
Fatty acids 20:4, *M (SD)*	0	62 (35)	58 (38)	59 (34)	73 (31)	NS
	12	63 (36)	78 (23)	56 (37)	64 (43)	NS
	24	70 (33)	83 (18)	63 (37)	73 (32)	NS
Sum of n-3 fatty acids, *M (SD)*	0	34 (37)	25 (35)	37 (38)	36 (39)	NS
	12	41 (38)	59 (40)	34 (36)	34 (37)	NS
	24	33 (35)	44 (37)	27 (34)	34 (37)	NS
Fatty acids 18:3, *M (SD)*	0	39 (22)	39 (26)	39 (21)	37 (22)	NS
	12	47 (22)	50 (20)	44 (20)	51 (30)	NS
	24	42 (18)	49 (17)_a_	37 (14)_ab_	48 (26)_b_	NS
Sum of long n-3 fatty acids, *M (SD)*	0	37 (48)	30 (46)	41 (49)	36 (50)	NS
	12	21 (41)	20 (41)	16 (37)	35 (49)	NS
	24	24 (42)	20 (41)	25 (43)	25 (44)	NS
EPA fatty acids 20:5, *M (SD)*	0	73 (44)	66 (49)	73 (44)	80 (40)	NS
	12	68 (46)	53 (52)	67 (47)	89 (30)	NS
	24	73 (44)	66 (49)	73 (44)	80 (40)	NS
DPA fatty acids 22:5, *M (SD)*	0	57 (47)	46 (51)	58 (45)	72 (47)	NS
	12	53 (43)	54 (47)	49 (48)	61 (49)	NS
	24	54 (47)	63 (47)	47 (47)	62 (49)	NS
DPA fatty acids 22:6, *M (SD)*	0	84 (32)	75 (43)	89 (23)	82 (34)	NS
	12	84 (29)	86 (26)	81 (33)	89 (17)	NS
	24	89 (28)	93 (26)	84 (31)	95 (15)	NS
Sum of trans fatty acids, *M (SD)*	0	6 (12)	5 (13)	7 (14)	4 (6)	NS
	12	12 (18)	9 (17)	15 (20)	5 (5)	NS
	24	9 (15)	7 (8)	9 (11)	12 (28)	NS
Cholesterol, *M (SD)*	0	63 (18)	62 (19)	63 (19)	66 (14)	NS
	12	72 (18)	76 (17)	69 (20)	76 (17)	NS
	24	72 (17)	75 (12)	71 (19)	70 (21)	NS
Retinol equivalents, *M (SD)*	0	43 (24)	43 (22)	44 (25)	39 (23)	NS
	12	48 (26)	56 (29)	45 (24)	46 (27)	NS
	24	50 (21)	52 (11)	49 (121)	48 (31)	NS
Retinol, *M (SD)*	0	26 (22)	24 (17)	26 (24)	31 (23)	NS
	12	34 (24)	43 (27)	29 (22)	34 (24)	NS
	24	33 (22)	36 (13)	30 (23)	37 (30)	NS
Beta carotene, *M (SD)*	0	84 (16)	81 (17)	87 (14)	80 (19)	NS
	12	84 (21)	86 (15)	84 (20)	79 (29)	NS
	24	86 (16)	83 (18)	90 (13)	81 (19)	NS
Vitamin D, *M (SD)*	0	58 (25)	61 (29)	57 (24)	57 (23)	NS
	12	63 (25)	73 (19)	59 (26)	64 (26)	NS
	24	66 (23)	77 (14)_a_	59 (24)_a_	70 (23)	0.024
Vitamin E, *M (SD)*	0	55 (18)	52 (18)	59 (17)	47 (17)	NS
	12	62 (18)	68 (15)	60 (18)	60 (22)	NS
	24	61 (16)	64 (15)	60 (15)	57 (22)	NS
Vitamin K, *M (SD)*	0	53 (27)	54 (31)	56 (25)	46 (25)	NS
	12	62 (29)	65 (31)	60 (31)	62 (24)	NS
	24	63 (29)	73 (26)	57 (28)	64 (32)	NS
Thiamin, *M (SD)*	0	47 (16)	46 (16)	48 (17)	42 (16)	NS
	12	54 (19)	61 (20)	52 (18)	53 (19)	NS
	24	55 (16)	55 (19)	54 (15)	57 (16)	NS
Riboflavin, *M (SD)*	0	41 (17)	44 (17)	40 (18)	39 (12)	NS
	12	47 (19)	51 (22)	45 (19)	48 (17)	NS
	24	47 (17)	49 (21)	45 (15)	51 (19)	NS
Vitamin C, *M (SD)*	0	78 (23)	85 (15)	80 (23)	66 (30)	NS
	12	83 (20)	87 (16)	82 (21)	80 (20)	NS
	24	85 (19)	86 (17)	85 (20)	81 (21)	NS
Niacin, *M (SD)*	0	63 (16)	64 (15)	63 (17)	62 (17)	NS
	12	68 (19)	75 (19)	64 (20)	66 (18)	NS
	24	69 (14)	72 (12)	68 (13)	66 (20)	NS
Niacin equivalents, *M (SD)*	0	58 (15)	59 (16)	57 (15)	56 (15)	NS
	12	62 (18)	69 (18)	59 (18)	61 (16)	NS
	24	63 (13)	65 (12)	62 (13)	63 (17)	NS
Vitamin B6, *M (SD)*	0	63 (15)	67 (14)	63 (16)	59 (17)	NS
	12	68 (17)	77 (14)_a_	65 (18)	66 (15)_a_	NS
	24	69 (13)	71 (13)	68 (12)	72 (16)	NS
Vitamin B12, *M (SD)*	0	55 (24)	58 (25)	53 (25)	56 (21)	NS
	12	59 (23)	59 (26)	57 (23)	66 (21)	NS
	24	64 (22)	71 (19)	59 (23)	67 (20)	NS
Phosphorous, *M (SD)*	0	41 (13)	44 (16)	41 (13)	38 (10)	NS
	12	47 (17)	52 (20)	44 (15)	48 (15)	NS
	24	45 (14)	46 (15)	43 (13)	50 (15)	NS
Folate, *M (SD)*	0	54 (17)	57 (16)	54 (16)	49 (19)	NS
	12	59 (19)	63 (22)	56 (19)	61 (15)	NS
	24	58 (16)	56 (15)	58 (15)	62 (20)	NS
Iodine, *M (SD)*	0	65 (21)	64 (25)	70 (21)_a_	52 (11)_a_	0.018
	12	70 (19)	73 (18)	72 (21)	61 (17)	NS
	24	70 (21)	65 (20)_a_	69 (24)_b_	82 (10)_ab_	NS
Iron, *M (SD)*	0	48 (15)	47 (16)	49 (14)	44 (14)	NS
	12	53 (19)	63 (21)_a_	47 (17)_a_	55 (16)	0.026
	24	51 (16)	53 (13)	50 (14)	49 (26)	NS
Calcium, *M (SD)*	0	22 (13)	24 (12)	24 (14)	17 (5)	NS
	12	28 (15)	28 (19)	27 (14)	30 (16)	NS
	24	29 (16)	27 (16)	29 (17)	31 (14)	NS
Potassium, *M (SD)*	0	60 (14)	63 (14)	61 (13)	53 (15)	NS
	12	64 (14)	69 (15)	62 (15)	65 (13)	NS
	24	64 (14)	64 (17)	63 (12)	67 (14)	NS
Magnesium, *M (SD)*	0	45 (13)	49 (14)	45 (12)	38 (13)	NS
	12	51 (17)	59 (19)	47 (15)	51 (16)	NS
	24	47 (13)	50 (12)	44 (12)	52 (19)	NS
Sodium, *M (SD)*	0	47 (16)	47 (16)	50 (17)	39 (15)	NS
	12	54 (17)	58 (17)	51 (17)	57 (15)	NS
	24	53 (15)	50 (14)	52 (15)	59 (17)	NS
Selenium, *M (SD)*	0	65 (16)	65 (20)	66 (16)	64 (14)	NS
	12	69 (18)	71 (20)	67 (17)	70 (16)	NS
	24	71 (17)	70 (13)	70 (19)	74 (12)	NS
Zink, *M (SD)*	0	49 (14)	49 (16)	50 (14)	45 (13)	NS
	12	52 (17)	60 (17)	48 (15)	53 (16)	NS
	24	51 (16)	53 (15)	49 (15)	56 (17)	NS

*Note*. Paleolithic Diet Fraction (PDF) for macro- and micronutrients by diet allocation, based on all 57 participants who completed the study per protocol (from 73 randomized participants) with complete dietary data. Group comparisons were conducted using one-way ANOVA, or Kruskal-Wallis H test, as appropriate. Means sharing the same subscript are significantly different from each other

NS = Not Significant (*p* > .05)

**Table 4:** Paleolithic Diet Fraction (PDF) for energy and weight, and Paleolithic Diet Score (PDS) at baseline, 12 months, and 24 months

Variable	Time (months)	*r*_s_ (55)	95% CI Lower	95% CI Upper	*p*
PDF for Energy vs. PDS	0	0.69	0.52	0.81	< 0.001
PDF for Energy vs. PDS	12	0.75	0.60	0.85	< 0.001
PDF for Energy vs. PDS	24	0.51	0.28	0.68	< 0.001
PDF for Weight vs. PDS	0	0.65	0.46	0.78	< 0.001
PDF for Weight vs. PDS	12	0.68	0.51	0.80	< 0.001
PDF for Weight vs. PDS	24	0.38	0.13	0.59	0.004
PDF for Energy vs. PDF for Weight	0	0.81	0.69	0.88	< 0.001
PDF for Energy vs. PDF for Weight	12	0.85	0.75	0.91	< 0.001
PDF for Energy vs. PDF for Weight	24	0.71	0.55	0.82	< 0.001
Δ PDF for Energy vs. Δ PDS	0–12	0.68	0.50	0.80	< 0.001
Δ PDF for Energy vs. Δ PDS	12–24	0.55	0.33	0.71	< 0.001
Δ PDF for Weight vs. Δ PDS	0–12	0.62	0.43	0.76	< 0.001
Δ PDF for Weight vs. Δ PDS	12–24	0.47	0.24	0.66	< 0.001
Δ PDF for Energy vs. Δ PDF for Weight	0–12	0.80	0.68	0.88	< 0.001
Δ PDF for Energy vs. Δ PDF for Weight	12–24	0.76	0.62	0.86	< 0.001

*Note*. Correlations between the Paleolithic Diet Fraction (PDF) for energy and weight, and Paleolithic Diet Score (PDS) at baseline, 12 months, and 24 months, based on all 57 participants who completed the study per protocol (from 73 randomized participants) with complete dietary data

Δ = Relative change (%)

There were no significant associations between absolute or relative change for PDS or PDF for energy and weight and relative change of cardiometabolic outcomes (*p =* .060-0.991). The sensitivity analysis in the form of an intention-to-treat analysis yielded similar results.

## Discussion

We found moderate to strong correlations between PDF and PDS, indicating their comparability [21] and strengthening the validity of both measures in assessing how closely a food intake aligns with a Paleolithic dietary pattern. The strongest correlations between PDF for energy and weight and PDS occurred at 12 months, possibly due to the largest spread of participant PDF and PDS at this time point. The increased spread at 12 months probably reflects dietary changes due to study intervention, which then decline towards the end of the study, mirroring the decreasing effect of dietary interventions often seen in longer-term follow-up [22]. The correlation was strongest between PDF for energy and PDS, which is unexpected since PDS in this study was calculated from dietary intake by weight. Calculating PDS from dietary intake by weight is in line with earlier studies in which PDS has been calculated from habitual food-consumption information in amounts and servings obtained from food frequency questionnaires [6, 7, 10, 14]. In an RCT involving 29 participants with ischemic heart disease, who were randomized to follow either a Paleolithic or Mediterranean-like diet, PDF for energy showed the strongest associations with cardiometabolic outcomes [16]. In contrast, in a randomized crossover study involving 13 participants with type 2 diabetes, who were randomized to eat a Paleolithic diet and a diabetes diet during two consecutive 3-month periods, PDF for weight had the strongest associations with cardiometabolic outcomes [9]. In theory, PDF for energy may be a more accurate measure of associations with outcomes when applied to less energy-dense Paleolithic foods, such as fruits, whereas the PDF for weight may be more accurate for more energy-dense Paleolithic foods, such as meat. When comparing the daily intake of food groups between these two studies, the 29 participants with ischemic heart disease had the highest daily intake of fruits, while the 13 participants with type 2 diabetes had the highest intake of meat products and eggs. The differences in dietary patterns may have contributed to why PDF for energy and weight had the strongest associations with cardiometabolic outcomes in their respective studies [9, 16]. Although the differences in associations with cardiometabolic outcomes between PDF for energy and weight were relatively small in both this study and in the two RCTs mentioned [9, 16], the results highlight the importance of calculating both PDF for energy and weight, whenever possible, to enhance the accuracy of PDF assessments and associations. In the Malmö Diet and Cancer Study (MDCS), a prospective cohort study involving 24,104 participants, only the PDF for weight could be specified. In contrast, only a measure similar to PDF for energy, the Paleo ratio, was assessed in a diet intervention in which a higher Paleo ratio was associated with improvements in cardiometabolic outcomes in patients with type 2 diabetes [8].

No significant associations were observed between changes in PDS, PDF (for energy and weight), and relative changes in cardiometabolic outcomes throughout the study. These findings align with the original study, which similarly reported no significant associations between dietary interventions and outcomes. Factors such as a small sample size and high variability in outcomes may have contributed to the lack of significant associations with cardiometabolic changes. Further, the absolute differences in PDF units between dietary interventions were small (0–9%) at baseline, 12 months, and 24 months, in contrast to previous studies that reported larger differences (27–44%) between the Paleolithic, Mediterranean-like, and diabetes diet interventions [9, 16]. Similarly, the absolute differences in PDS between dietary interventions were also small at baseline, 12 months, and 24 months (0–2 units).

The absolute and relative changes in PDF were greater than those in PDS across all groups during the study. These findings suggest that PDF might be a more sensitive measure than PDS for evaluating dietary changes, at least when the dietary changes are of the order of magnitude as in this study. Still, the PDFs for the dietary intervention ‘no grain’ were 43–57% and markedly lower compared to the PDFs for a Paleolithic diet in previous studies, which were 77–86% [9, 16]. At 12 months, the average daily intake of cereals in the ‘no grain’ groups was 142 g, compared to 16 g and 21 g in the Paleolithic diet groups reported in previous studies [9, 16, 19]. The significant disparity in daily cereal intake between the ‘no grain’ and Paleolithic diet groups may contribute to the substantial difference in PDF between the groups. Furthermore, the relatively unchanged PDFs from baseline to 12 months in the ‘no grain’ groups, despite a 43% reduction in cereal grains and a 32% reduction in whole grains, indicates a correlated increase in the consumption of other non-Paleolithic food groups [19].

Although the PDF for energy was only 40%, the PDFs for most micronutrients exceeded 50%, with the highest percentages observed for vitamin B12, vitamin C, and beta-carotene. These findings align with previous research and suggest a relatively high micronutrient density in the Paleolithic diet [1, 10, 24]. The only micronutrients with lower PDFs were calcium, magnesium, retinol, phosphorus, and riboflavin (Table 3). This finding aligns with the MDCS, where the correlations between quintiles of the PDF and absolute intakes of micronutrients showed negative trends only for calcium, retinol, and riboflavin [10]. A critique of the Paleolithic diet is its relatively low calcium content. However, research by Boers et al. demonstrated that reduced calcium intake is offset by lower urinary calcium excretion, effectively maintaining calcium homeostasis [23]. Additionally, the Paleolithic diet’s low-salt, high-protein, and alkalizing properties, further supports its potential benefits for bone health [5].

The mean PDF at baseline was 36% for energy, and 45% for weight (Table 2), which is similar to previous study results for Mediterranean-like and diabetes diet [9, 24]. Additionally, similar PDFs were observed in the MDCS cohort [10] and a Paleolithic diet intervention study with 32 participants diagnosed with type 2 diabetes [8]. These comparisons highlight the shared characteristics and underscore how distinctly the standard Swedish diet, Mediterranean-like diet, and diabetes diet differ from the Paleolithic diet in terms of food group composition.

### Strengths

The strengths of this study include its longer duration and larger sample size compared to previous dietary intervention studies on the PDF [8, 9, 24], which enhance the validity of comparisons between the PDF and PDS. Furthermore, the inclusion of four-day weighed food records collected at baseline, 12 months, and 24 months provides a rigorous and comprehensive assessment of dietary intake over time. Another strength of this study is the use of the Swedish National Food Agency’s Food Database for calculating the PDF and analyzing food composition. This database is updated regularly, operates independently of researchers, and is a locally well-established resource with high credibility, ensuring the reliability of the dietary data [20].

### Limitations

A key limitation of the study is that PDS was calculated using four-day weighed food records rather than a food frequency questionnaire, which reduces comparability with previous research. The lack of significant associations between PDS or PDF and cardiometabolic outcomes was likely influenced by insufficient participant recruitment and an underestimation of variability in outcome changes, as previously surmised [19]. Furthermore, the participants’ dietary changes during the study only translated into small changes in PDF and PDS, which further reduced statistical power. A post hoc power estimate showed that at least one thousand individuals would have been needed to detect a significant association between changes in PDF and PDS and waist circumference. Fasting plasma glucose, HbA1c, systolic blood pressure, and diastolic blood pressure were excluded from association analysis due to changes in medication among diabetic and hypertensive participants at unknown times during the study. Therefore, potential associations between PDF and PDS and glucose metabolism and blood pressure may have been overlooked. The predominantly female study population limits the representativeness of the findings. Finally, the post hoc nature of these analyses necessitates cautious interpretation and underscores the need for validation in future studies.

### Future research

Future research should explore comparisons between PDF and other established diet-quality indices, such as the Mediterranean Diet Score, in large-scale cohort studies. Such comparative analyses are important for determining their relative contributions to cardiometabolic health. Moreover, future studies should aim to include larger sample sizes and design dietary interventions that cause more pronounced changes in PDF, enabling a more accurate evaluation of its associations with glucometabolic outcomes.

Moreover, PDF could be applied directly in dietary intervention studies. Participants could be instructed to modify their diets to achieve predefined PDF targets, allowing evaluation of both the methods of dietary adaptation and the resulting health impacts. Such research could offer insights into the practicality and effectiveness of utilizing PDF as a framework for dietary recommendations and its potential role in future dietary guidelines.

## Conclusion

There were moderate to strong correlations between PDF and PDS and no associations between PDF or PDS and cardiometabolic outcome measures, with the latter result possibly due to only modest changes in diet and an absence of outcome effects.

## Data Availability

The dataset analyzed during the current study is available from the corresponding author upon reasonable request.

## Abbreviations

BIA: Bioelectrical impedance analysis
BMI: Body mass index
FPG: Fasting plasma glucose
HbA1c: Glycated hemoglobin
HDL: High-density lipoprotein
LTP: Leisure time physical activity
non-HDL: Non-high-density lipoprotein
OGTT: Oral glucose tolerance test
PDF: Paleolithic diet fraction
PDS: Paleolithic diet score
RCTs: Randomized controlled trials
SED: Sedentary time
SBP and DBP: Systolic and diastolic blood pressure
